# Altitude and Erythropoietin: Comparative Evaluation of Their Impact on Key Parameters of the Athlete Biological Passport: A Review

**DOI:** 10.3389/fspor.2022.864532

**Published:** 2022-06-30

**Authors:** Jonas J. Saugy, Tania Schmoutz, Francesco Botrè

**Affiliations:** ^1^Institute of Sport Sciences, University of Lausanne (ISSUL), Lausanne, Switzerland; ^2^Research and Expertise in anti-Doping Sciences (REDs), University of Lausanne, Lausanne, Switzerland

**Keywords:** anti-doping, athlete biological passport (ABP), blood, erythropoietin, altitude

## Abstract

The hematological module of the Athlete's Biological Passport (ABP) identifies doping methods and/or substances used to increase the blood's capacity to transport or deliver oxygen to the tissues. Recombinant human erythropoietin (rhEPOs) are doping substances known to boost the production of red blood cells and might have an effect on the blood biomarkers of the ABP. However, hypoxic exposure influences these biomarkers similarly to rhEPOs. This analogous impact complicates the ABP profiles' interpretation by antidoping experts. The present study aimed to collect and identify, through a literature search, the physiological effects on ABP blood biomarkers induced by these external factors. A total of 43 studies were selected for this review. A positive correlation (R^2^ = 0.605, r = 0.778, *p* < 0.001) was identified between the hypoxic dose and the increase in hemoglobin concentration (HGB) percentage. In addition, the change in the reticulocyte percentage (RET%) has been identified as one of the most sensitive parameters to rhEPO use. The mean effects of rhEPO on blood parameters were greater than those induced by hypoxic exposure (1.7 times higher for HGB and RET% and 4 times higher for hemoglobin mass). However, rhEPO micro-doses have shown effects that are hardly distinguishable from those identified after hypoxic exposure. The results of the literature search allowed to identify temporal and quantitative evolution of blood parameters in connection with different hypoxic exposure doses, as well as different rhEPOs doses. This might be considered to provide justified and well-documented interpretations of physiological changes in blood parameters of the Athlete Biological Passport.

## Introduction

The athlete biological passport (ABP) was proposed for the first time in the early 2000s. The primary aim was to monitor and record a selection of hematological variables in a longitudinal follow-up, with the aims of eliminating potential interindividual variability and focusing on intraindividual variations by establishing the athlete as his or her own reference (Sottas et al., 2010, 2011; WADA, 2021). The passport represents an evolution in the paradigm of anti-doping methods: from the direct detection of forbidden substances and/or their metabolites to an indirect, multiparametric method. Its hematological module considers 14 variables regrouped into two primary biomarkers, which are the hemoglobin concentration (HGB, in g/dL) and an Off-hr score [Off-hr = (Hb) (g/L−60 x √Reticulocytes percentage) (Ret%)]. The reticulocyte percentage, which is calculated as the absolute reticulocyte count (per μL) divided by the red blood cell count (per μL) as a percentage, and the Abnormal Blood Passport Score (ABPS) represent the secondary biomarkers. A primary biomarker is defined as specific to doping where secondary markers provide supporting evidence of doping alone or in combination with other secondary markers (WADA, 2021). The application of Bayesian inference is used to redefine a potential doping activity (the cause) that would have modified one or more selected biomarker(s) (the effect) (Sottas et al., 2010; Aikin et al., 2020).

When a potential abnormal evolution of the hematological parameters is recorded by the ABP, a subjective evaluation of data by scientific experts is required (Schumacher and D'onofrio, 2012). Some of these alterations might be induced by several confounding factors that may alter the natural variability of a physiological parameter and thereby influence the sensitivity and/or specificity of the protocol used to monitor the selected biomarker (Saugy et al., 2014b). Thus, those factors might exert a significant effect on the evaluation process used by the experts. Confounding factors of the ABP hematological module have been identified in several studies, including chronic training, acute training, hyperthermia [e.g., sauna (Stanley et al., 2015), heated chambers, and local hyperthermia protocol], hypothermia (e.g., cryogenic protocols and cold acclimation), heterogeneous factors (e.g., age, sex, and genotype) (Sottas et al., 2011), various disorders or illnesses (e.g., genetic illness, chronic or acute sickness, and anemia), the environmental condition of the test (e.g., circadian cycle, level of hydration, and body position), hypoxic training [e.g., hypobaric or normobaric hypoxia, blood flow restriction (BFR), intermittent exposure, and chronic exposure], and, finally, doping practices. A more exhaustive overview of these confounding factors has been available in a recent systematic review (Krumm and Faiss, 2021).

Furthermore, among all the potential confounding factors mentioned, the latter two (i.e., hypoxic exposure and doping practices) might exert significant effects on the blood variables, and differentiating whether those effects are derived from one or the other factor or even from the combination of both is sometimes difficult.

Physiologically, hypoxic conditions lead to the activation of hypoxia-inducible transcription factors (HIFs) due to oxygen decrease in the cellular environment; Hypoxia-inducible factor activating agents are also included in the World Anti-Doping Ageny (WADA) prohibited list. This decrease results in increased production of Erythropoietin [EPO, (Kasperska and Zembron-Lacny, 2020)]. The main effect is an increased production of red blood cells, stimulated by erythropoiesis, to balance the lack of oxygen uptake (Lobigs et al., 2018b). The associated changes in HGB, RET%, and thus Off-hr resulting from hematological adaptation to the hypoxic condition may result in the individual limits of the ABP being exceeded (Schumacher and D'onofrio, 2012). Altitude training is then considered one of the main confounding factors in the variability of the ABP model (Sanchis-Gomar et al., 2011, 2014). Therefore, training at altitude must be considered when analyzing the evolution of hematological parameters (Saugy et al., 2014b). Moreover, although potential effect on improving the hematological variables is still being questioned (Ploszczyca et al., 2018), many methods of hypoxic training designed to stimulate the production of EPO and, thereby, increase the production of erythrocytes exist (Wilber, 2007; Millet et al., 2013).

However, erythrocyte production is also improved by blood doping, with recombinant human EPO (rhEPO) (Thomsen et al., 2007; Clark et al., 2017). Indeed, physiological changes induced by rhEPO injection exceeded the magnitude of the changes associated with natural altitude exposure by approximately 100% (Parisotto et al., 2001). Various studies have been conducted to better understand the effects of rhEPO on physiological parameters and performance. Some clinical trials indicated that optimal results were obtained with injections of rhEPO at least every 2–3 days to maintain a substantially elevated rate of erythropoiesis. Since then, different rhEPO administration protocols of low and high doses (10 to 350 IU/kg body weight) during short or long periods (10 days to 6 weeks) have appeared throughout the scientific literature (Thomsen et al., 2007; Bejder et al., 2016a,b; Martin et al., 2016; Clark et al., 2017; Leuenberger et al., 2017; Wang et al., 2017; Haile et al., 2019; Heuberger et al., 2019; Marchand et al., 2019). If athletes may have initially used normal to high doses of rhEPO (30–350 IU/kg), they quickly switched to low doses (~10–25 IU/kg) or even micro-doses [5–10 IU/kg (Lasne et al., 2009)] after the emergence of direct EPO-tests (Lasne and De Ceaurriz, 2000) and the development and optimization of indirect detection methods (Sottas et al., 2010; Salamin et al., 2018). The use of micro-doses also reduces the sensitivity of the athlete biological passport (Ashenden et al., 2011). Because of this limited time of detection, indirect tests to identify rhEPO using blood markers of altered erythropoiesis, such as the ON-OFF score or the ABP, were developed (Parisotto et al., 2000; Gore et al., 2003; Sharpe et al., 2006; Robinson et al., 2011).

Thus, the aim of the present study was to assemble and identify, through a literature search, physiological effects on key blood biomarkers of the ABP induced by various hypoxic exposures and rhEPO injections. The objective is then to provide key information about understanding, interpreting, and differentiating how the ABP hematological module is potentially affected by those external factors.

## Methods

### Data Sources and Research Algorithms

Since the literature search targeted the confounding factors of hypoxic training and exposure and rhEPO doping, the selected keywords needed to match those factors and the effects on the four ABP biomarkers described above. The databases PubMed and Google Scholar were used for the first phase of the literature search, which was conducted between December 2020 and January 2021. A few search algorithms were created using keywords that appeared in titles and abstracts, and Boolean connectors, such as “AND” and “OR”. For the confounding factor of altitude, the final algorithm used was: (altitude OR hypoxia) AND (athlete biological passport OR passport OR hematological module OR OFF-score OR RET OR ABPS) AND (erythropoiesis OR hemoglobin OR hemoglobin mass OR hematocrit OR red blood cell volume OR blood volume OR plasma volume OR blood parameter). Another algorithm was applied for the confounding factor of EPO doping: (EPO OR rEPO OR rhEPO OR rHuEPO OR recombinant erythropoietin OR ESA) AND (athlete biological passport OR passport OR hematological module). A total of 404 (253 for altitude and 151 for EPO) articles were retrieved from the two databases.

### Study Selection and Inclusion Criteria

**Figure 1:**
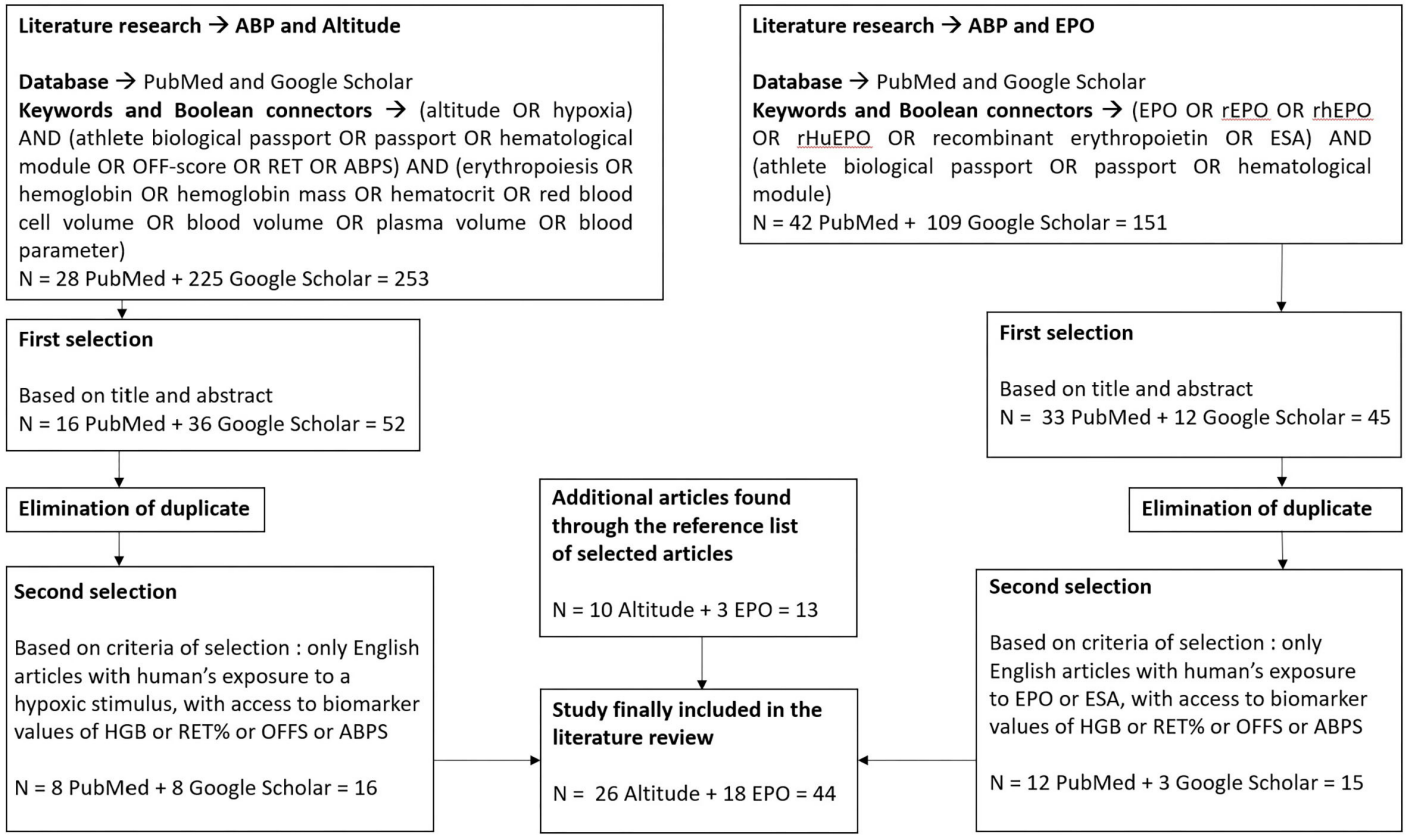
Diagram of the literature search process. HGB, concentration of hemoglobin; RET%, reticulocyte percentage; Off-hr, stimulation index Off-score; ABPS, abnormal blood profile score; EPO, erythropoietin; rhEPO, recombinant human erythropoietin; ESA, erythropoiesis-stimulating agent; *n*, number of articles present at each step.

Based on titles and abstracts, 97 articles were first selected by two independent reviewers. Then, duplicates were eliminated, and the remaining articles were examined and selected based on several inclusion criteria: articles must be written in English, analyzed human subjects who were exposed to either hypoxia or rhEPO stimulus, and hematological data, such as HGB, RET%, Off-hr, and/or ABPS were available. At the end of this second step, 31 articles were selected. Additional pertinent papers (*n* = 13) found by searching in the bibliography of the selected articles were added to the final selection, which included 44 articles. Figure 1 outlines the literature search procedure.

### Outcome Parameters

The second phase of the literature search consisted of extracting all the data recorded in the 44 articles. Information about the number of subjects in intervention groups and control groups, fitness status of the subjects, type of sport practiced by the participants, altitude mode, hypoxic condition, altitude, time of exposure, hypoxic dose, number of measures, EPO doses, frequency of injections, supplement (iron, vitamin or hyperhydration), HGB, RET%, Off-hr, Hb_mass_, serum-EPO (s-EPO), and abnormal ABP markers was collected.

## Results and Discussion

### Altitude and Blood Parameters

Any comparison between two or more studies is difficult due to the diversity of the proposed protocols {fitness status of the participants, sex, altitude mode, type of hypoxic stimulus [i.e., hypobaric hypoxia (HH) or normobaric hypoxia (NH), altitude (m), duration of exposure, number of measures, etc.]}. The parameter “kilometer hours” (km.h) established by Garvican-Lewis et al. (2016) was calculated for each study after considering hypoxic exposure as a confounding factor in the literature search to more easily compare the hypoxic dose administered in each protocol described in the studies. This model considers the roles of both exposure time and the altitude level. This metric is simply defined by the quotient of altitude exposure (m) divided by 1,000, multiplied by the total duration of the exposure (h): (altitude (m)/1,000) x hours (h). When a study examined more than one altitude, the sum of each different km.h value was calculated.

Table 1 illustrates the results obtained during the literature search involving different types of hypoxic exposures as confounding factors. The results showed three different levels of hypoxic dose. The smaller doses, ranging between 37 (Kasperska and Zembron-Lacny, 2020) and 320 km.h (Abellan et al., 2007), were present in intermittent hypoxic exposure (IHE) and interval hypoxic training (IHT) protocols. The Live High–Train Low (LHTL) camps proposed larger hypoxic doses, ranging from 520 (Saugy et al., 2016b) to 1,250 km.h (Wehrlin and Hallen, 2006). The largest hypoxic doses were logically observed during Live High–Train High (LHTH) protocols. Without considering the paper by Saugy et al. (2016a), where the time of exposure was only ~26 h (hypoxic dose of 90 km.h), the hypoxic doses in these protocols ranged from 1,300 (Wachsmuth et al., 2012) to 2,320 km.h (Siebenmann et al., 2015). The variations in the hemoglobin percentage ranged from −8.2% (Garvican-Lewis et al., 2014) to 19.6% (Beidleman et al., 2017) (average of 6.6 ± 8% based on all the altitude studies and 7 ± 3.9% after excluding the “other hypoxic exposures” studies–see Table 1). In 10 studies, the increase in HGB [%] was not significant at the end of the hypoxic exposure. The variations in the reticulocyte percentage ranged from −35 to 350% (average of 29 ± 102% when considering all studies, 70 ± 140% during hypoxic exposure, −6 ± 37.4% post hypoxic exposure values). The hemoglobin mass showed a significant increase in nine studies, with an average increase of 4.7 ± 1.6% (min, 2.6%; max, 7.5%). The analysis of s-EPO levels showed an increase ranging from 46.5% after the 1st day of hypoxic exposure [LHTL, 2,650–3,000 m, NH, (Ashenden et al., 2003)] up to 439% 3 h after the final hypoxic exposure on Day 26 [IHE, 5,500 m, NH, (Abellan et al., 2007)]. After altitude exposure, s-EPO levels decreased compared to the baseline, with values ranging from −25.7% after 18 days of the LHTL camp (living altitude, 2,250 m; training altitude, < 1,200 m) to −45% after 4 weeks of IHT, with an *F*_*i*_*O*_2_ of 10 to 12% (corresponding to ~5,800–4,500 m).

**Table 1:** Summary of the main parameters collected in the 26 altitude studies of the literature search.

**N**°****	**Autor + year**	**Subjects-activity [n]**	**Altitude [m]**	**Time of exposure**	**Hypoxic dose [km.h]**	**Delta HGB [%]**	**Delta RET [%]**	**Delta Off-hr [%]**	**Delta Hbmass [%]**	**Abnormal ABP markers [%]**
***Intermittent Hypoxic Exposure***										
1	Abellan et al. (2007)	8 (triathlon)	4,000–5,500	3 h/d during 5 d/w during 4 weeks	320	NS	NS	-	-	-
2	Gore et al. (2006)	11 (swimming, running)	40,00–5,500	3 h/d during 5 d/w during 4 weeks	300	NS	NS	-	NS	-
3	Julian et al. (2004)	7 (running)	F_i_O_2_ = 0.12 – 0.10	70 min/d, 5 times/week during 4 weeks	115	4.1	−31.6 (P10)	-	-	-
4	Kasperska and Zembron-Lacny (2020)	6 (wrestling)	2,500–4,500	1 h/d during 10 days	37	NS	350 (D10)	-	-	-
5	Fernández-Lázaro et al. (2020)	12 (athletics)	4,000 – 5,000+	90 min/day during 4 weeks	200	NS	9.6 (P0)	-	-	-
***Live High – Train High***										
6^a^	Ashenden et al. (2003)	19 (cycling)	2,690	26–31 days	2,000	6.9	NS	-	-	-
7	Bejder et al. (2020)	27 (running, cycling)	2,320	4 weeks	1,560	14.6	46 (D15)	-	-	31
		12 (running, cycling)				8.3	-			-
8	Bonne et al. (2014)	10 (swimming)	2,130 – 3,094	3–4 weeks	1,600	NS	−29 (P7)	21.3	-	40
9	Garvican et al. (2012)	8 (cycling)	2,760	18–22h/d, during 3 weeks	1,400	-	20.4 (D12)	-	3.5	-
10	Gore et al. (1998)	8 (cycling)	2,690	31 days	2,000	NS	−62 (P21)	-	NS	-
11^a^	Levine and Stray-Gundersen (1997)	13	2,500	4 weeks	1,680	8.7	-	-	-	-
12	Siebenmann et al. (2015)	9	3,454	28 days	2,320	14.6	-	-	5.26	-
13	Sutehall et al. (2019)	14 (running)	2,800	27 days	1,815	-	-	-	-	64
14	Wachsmuth et al. (2012)	31 (swimming)	2,320 (3 camps)	Between 18 and 27 days	1,300	3.4	−7.8 (P1)	11.6	7.5	-
15	Young et al. (2019)	17	4,300	22 days	2,270	10.7	-	-	-	-
***Live High – Train Low***										
6^b^	Ashenden et al. (2003)	6 (running)	2,100	altitude resident	-	NS	NS	-	-	-
6^c^	Ashenden et al. (2003)	24 (triathlon)	2,650–3,000	8–10h/d during 20–23 days	525	2.96	NS	-	-	-
16	Clark et al. (2009)	12 (cycling)	3,000	14 h/d during 3 weeks	880	-	-	-	3.3	-
17	Garvican-Lewis et al. (2018)	34 (endurance)	3000	14 h/d during 21 nights	880	4.1	-	8.5	-	32.3
11^b^	Levine and Stray-Gundersen (1997)	13 (running)	2500	16–17 h/d during 4 weeks	1,175	11.3	-	-	5.3	-
18	Lobigs et al. (2018a)	34 (endurance)	3,000	14 h/d during 21 days	880	6	-	-	-	26.5
19	Saugy et al. (2014a)	13 (triathlon)	2,250	12 h/d (NH) or 17 h/d (HH) during 18 days	675	4.2	23.2 (P1)	-	3.4	-
		14 (triathlon)			495	NS	21.6 (P1)		2.6	
20	Saugy et al. (2016b)	10 (triathlon)	2,250	13 h/d (NH) or 17 h/d (HH) during 18 days	700	2.4	ns	-	-	-
		6 (triathlon)			520	4.3				
21	Voss et al. (2020)	10 (running)	3,000–5,400	6 h/d during 14 days	792	6.1	-	-	-	30
			2,400–3,000	10 h/d during 14 days		-				-
22	Wehrlin and Hallen (2006)	10 (endurance)	2,500	18–19 h/d during 24 days	1,250	NS	43.4 (P8)	-	5.5	-
***Other Hypoxic Exposures***										
23	Beidleman et al. (2017)	393	2,500	Between 2 h and 7 days of ascent and acclimation	5–420	14.6	-	-	-	-
			3,000		6–504	15.4				
			3,500		7–588	16.8				
			4,000		8–672	18.2				
			4,500		9–756	19.6				
24	Garvican-Lewis et al. (2014)	9 (cycling)	1,146 – 4,120 (average 2,187)	14 days stage racing	780	−7	28 (D10)	-	6	-
		9 (cycling)	1,146 – 4,120 (average 2,187)			−8.2	−35 (D15)		NS	
25	Saugy et al. (2016a)	13	3,450	26 h	90	5.8	NS	-	-	-
						NS	-			
26	Schumacher et al. (2015)	14 (cycling)	1,159–3,491 (average 2,496)	14 days stage racing	780	−7	-	-	-	20
		11 (cycling)				−8.2				8

*Only the highest HGB, RET%, Off-hr and Hbmass increases are presented. HGB, concentration of hemoglobin; RET%, reticulocyte percentage; Off-hr, stimulation index Off-score; Hbmass, hemoglobin mass; ABP, athlete biological passport*.

#### Hypoxic Dose

Different hypoxic doses were used across the selected studies. The smallest doses were used in the IHE and IHT protocols because they allowed only 1–33 h of hypoxic exposure at high altitudes from 10 days to 4 weeks (and in the case of 4 weeks, only 5 days per week) (Julian et al., 2004; Gore et al., 2006; Abellan et al., 2007). In LHTL studies, the alternation of moderate and high altitudes for training and living purposes enabled exposure to a larger hypoxic dose while conserving the intensity of training sessions. In comparison, the advantage of the LHTH method is the “nonstop” exposure time to the selected altitude, allowing exposure to maximal hypoxic doses but without the possibility of reaching maximal training intensities. In addition, the type of hypoxic stimulus plays an important role, as described by Saugy et al. (2014a), where HH and NH conditions resulted in different hypoxic exposures for the same duration (in days) of the training camp (774.75 vs. 515.7 km.h for HH and NH, respectively). For practical reasons, high hypoxic doses are difficult to achieve when using simulated hypoxia. Indeed, a confinement duration that is too long might induce complications, such as detrimental reductions in plasma volume (PV) (Siebenmann et al., 2012). In their study, Hauser et al. (2016) proposed a comparison of hematological data during an 18-day LHTL camp when hypoxic doses were matched, namely, on Day 13 of the HH condition (516 km.h) and on Day 18 of the NH condition (536 km.h) to overcome the issue of different hypoxia doses under NH and HH conditions. This difference in the number of days to pair the hypoxic doses is also identified in the literature search when LHTL and LHTH are compared. In the study by Saugy et al. (2014a), 18 days of training under HH condition were necessary for triathletes to reach the hypoxic dose of 675 km.h during an LHTL camp, and only 13 days were required for the cyclists in the study by Schumacher et al. (2015) to attain 779 km.h during their LHTH camp.

#### Hematological Adaptations During the 1st Days of Altitude Exposure

The first phenomenon observed in the literature, which is linked to altitude exposure and blood parameters of the ABP, was the increase in HGB levels during the first 2 days of exposure. Due to the decreased arterial oxygen content at altitude, Wachsmuth et al. (2012) identified the contraction of PV and hemoconcentration within 2 h of exposure to 3,600 m as a first short-term acclimation response of the body, as subsequently reported by other researchers (Siebenmann et al., 2015; Lobigs et al., 2018b). This finding has also been supported by Lobigs et al. (2018a), who showed that PV contraction might be considered as the initial primary confounder of HGB fluctuations. This hemoconcentration might be partially explained by a gap between fluid replacement requirements and the suppression of the renin-angiotensin axis and increases in urinary, respiratory, and transcutaneous fluid losses (Lobigs et al., 2018b). In HH, due to enhanced ventilation under hypoxic conditions and dryer air at high altitude, the respiratory loss of water is increased (Jain et al., 1980). Urinary water loss and the increase in exudation at altitude are also described in the literature as potential causes of this hemoconcentration (Clark et al., 2017). At this point, the effect on the ABP parameters is an increase in HGB levels without modification of the RET%, which will increase the Off-hr (Ashenden et al., 2003). The magnitude of PV contraction is described as proportional to hypoxic stress. Indeed, Beidleman et al. (2017) identified the evolution of PV during the 1st 7 days of passive exposure to different altitudes (from 2,500 m to 4,500 m) and showed a ~6% loss in PV after 24 h of exposure to 2,500 m. They also identified an additional ~1% loss in PV for every 500-m increase in elevation above 2,500 m. Nevertheless, the dehydration effect is less visible under the intermittent condition (LHTL for example) due to the oscillating nature of the protocols. Some studies did not report an initial hemoconcentration in all of the subjects, and substantial variability in the individual response to altitude was noted (Beidleman et al., 2017; Lobigs et al., 2018b). Beidleman et al. (2017) also described interindividual variability in the results, where large differences were observed for the effect of the duration at altitude on HGB.

Another early mechanism of adaptation to the hypoxic environment is the increase in s-EPO levels. In healthy subjects, endogenous EPO levels increase exponentially when blood hemoglobin concentrations decrease. An increase in s-EPO levels is well established directly proportional to hypoxia levels and peripheral oxygen saturation (*S*_*p*_*O*_2_) diminution (Haase, 2010). While normal values of s-EPO are ~6–32 IU/l, they may be multiplied by a factor of 3 under hypoxic conditions and could increase up to 10,000 IU/l in patients with a severe blood disease such as, anemia (Jelkmann, 2016). Because of the decreases in inspired oxygen partial pressure (P_i_O_2_), the plasma EPO level increases quickly during acclimatization to high altitude, with values peaking after 1–2 days. A dose-dependent relationship between the increase in the endogenous erythropoietin concentration and erythropoietic response has also been described by Perez-Ruixo et al. (2008). Several altitude training protocols have been shown to significantly increase s-EPO levels after the 1st to 3rd days/night at altitude, from the 1st dozen minutes to several hours of acute hypoxic exposure (Ploszczyca et al., 2018). The results of the literature search illustrate this change, with increases in s-EPO levels, ranging from 46.5% after the 1st day of hypoxic exposure [LHTL, 2,650–3,000 m, NH, (Ashenden et al., 2003)] to 439% 3 h after a final hypoxic exposure on Day 26 [IHE, 5,500 m, NH (Abellan et al., 2007)]. This latter study highlighted the interindividual responses in increased s-EPO levels and the effect of the protocol used on these changes. The s-EPO responses at different altitudes have been measured by Ge et al. (2002), who estimated that an altitude threshold at 2,100–2,500 m would produce a robust increase in s-EPO levels. This study also illustrated the interindividual variability in the increased s-EPO levels under hypoxic conditions. The range of s-EPO variation at 3,000 m compared to the baseline was −40 to +400% after 24 h (Ge et al., 2002). The relationship between s-EPO levels and a possible increase in Hb_mass_ is complex. After the peak, the s-EPO level starts to decrease gradually, but remains above initial values for a few days to weeks. Changes in s-EPO levels become nonsignificant compared to baseline values after the 1st, 2^nd^, or 3rd week at altitude (Ploszczyca et al., 2018). After returning to the sea level, s-EPO levels decreased to baseline values immediately or after a few days. In some cases, the s-EPO level even decreased below the initial value (Ploszczyca et al., 2018).

#### Effect on Reticulocytes

After this primary phase of 1–2 days, chronic adaptation of the main hematological parameters usually occurred 8 days after the beginning of altitude exposure. The increase in endogenous EPO levels in the body will increase the production of reticulocytes as first effect. An increased RET% due to hypoxic stress was reported in many included studies. Compared to baseline levels, increased RET% was observed in the first 300 km.hr of altitude exposure within the meta-analysis by Lobigs et al. (2018b). The literature search highlighted increases, ranging from 20.4% [LHTH, 12th day of hypoxic exposure, 2,760 m, 795 km.h at this time point; Garvican et al. (2012)] to 350% [IHE, NH, at the 10th day of hypoxic exposure, 2,500–4,500 m, 37 km.h (Kasperska and Zembron-Lacny, 2020)]. However, changes in RET% are not always significant, as described in the study by Ashenden et al. (2003) where no change in RET% at the end of 3 weeks of an LHTL camp at 2,650 m (NH) was noted. An interpretation of this result might be that RET% already increased during the camp without a blood test to confirm it, and it returned to the baseline at the end of the camp.

If RET% was increased by altitude exposure, normal endurance training would also increase the level of this biomarker. In fact, a 2–4% increase in RET% was observed in the control groups of the altitude training studies during the 30–70 days of protocol. Thus, an absolute change in RET% of ~2–4% might reflect normal changes due to training and, perhaps, not due to altitude exposure. In addition, no significant increased RET% under 9.6% [IHE exposure, NH, 90 min per day for 4 weeks at 4,000–5,000 m (Fernández-Lázaro et al., 2020)] was observed in the included literature. Finally, after the first 300 km.h of hypoxic dose, RET% levels return to the baseline, as the physiological adaptations to the hypoxic environment are sufficient to meet the oxygen transport needs by increasing the red cell mass (Reynafarje et al., 1959).

#### Evolution of Hemoglobin Concentration and Mass

**Figure 2:**
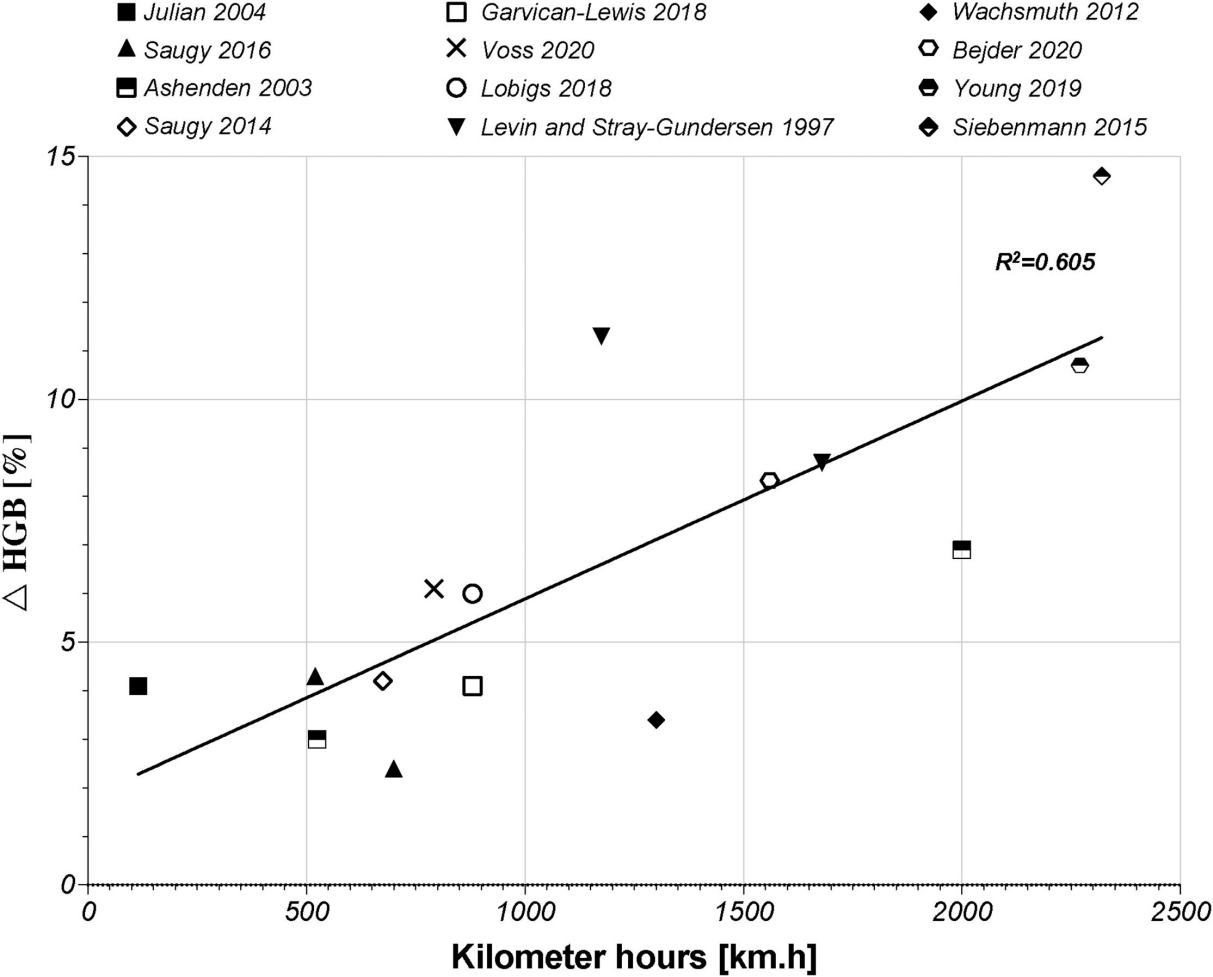
Graphic of the linear correlation between HGB (g/dl) and hypoxic dose (km.h) according to 12 studies of the literature search. HGB, hemoglobin concentration.

Subsequently to the RET% increase, induced by the increase in s-EPO levels, an actual increase (i.e., not due to PV shifts and hemoconcentration) in HGB and Hb_mass_ will occur. Regarding HGB, 22 studies retrieved in the literature search highlighted slight increases in HGB, ranging from 2.4% (Saugy et al., 2016b) up to 14.6% (Siebenmann et al., 2015). Interestingly, the largest increase in HGB was observed in the study that proposed the highest hypoxic dose [2,320 km.h in the study by Siebenmann et al. (2015)]. Our literature search supports this conclusion based on the study by Beidleman et al. (2017), who presented a progressive increase in HGB of 14.6–19.6% after 7 days at 2,500–4,500 m (420–756 km.h). According to a previous meta-analysis by Lobigs et al. (2018b), the increase in HGB levels is directly influenced by the increase in hypoxic dose. A previous study also showed that the HGB level is expected to peak in the final week of altitude exposure (Siebenmann et al., 2015). This conclusion is illustrated by the relation between the increase in HGB [%] and the hypoxic dose [km.h]. In fact, 60% of the increase in HGB levels might be explained by the change in the hypoxic dose (Figure 2, R^2^ = 0.605, r = 0.778, *p* < 0.001).

The more time a subject spends at altitude, the greater the increase in his or her HGB level is. The studies by Beidleman et al. (2017), Garvican-Lewis et al. (2014), and Schumacher et al. (2015) were not included in Figure 2. The first study only used passive altitude exposure, which did not include physiological adaptation to physical exercises. The other two studies were designed during stage race conditions, which represent maximal efforts during 14 days of competition and, therefore, induced important hemodilution despite an increase in Hb_mass_. Notwithstanding this correlation, the HGB level did not always show a significant increase, or only scarce changes at the end of a hypoxic exposure. In the study by Wachsmuth et al. (2012), for example, the HGB level only showed a small increase of ~3.4% between baseline and final camp measures. Another finding noted in the literature is a negative correlation between the baseline HGB level and ulterior changes at altitude. This result might be interpreted as a lower possibility of increasing HGB levels in participants who already have high-baseline HGB levels (Lobigs et al., 2018b). As explained above, HGB levels depend on plasma volume shifts, which may increase the complexity of the interpretation of the results (Lobigs et al., 2018a). Indeed, Garvican-Lewis et al. (2014) illustrated that multiday stage racing at altitudes between 1,146 and 4,120 m induced significant hemodilution (17% in sea-level residents and 16% in altitude residents). The decreases in HGB levels observed at the end of the stage races were ~−7 and −8.2% in the sea level and altitude residents, respectively. However, the hemoglobin level did not decrease *per se*. In fact, sea-level residents showed an increase in Hb_mass_ of 6% induced by altitude exposure (Garvican-Lewis et al., 2014). These results highlighted the important effects of factors, such as altitude and physical stress, on PV shifts and the interpretation of variations in HGB levels. Therefore, correlation between changes in HGB [%] and the hypoxic dose should be interpreted carefully. Nevertheless, PV is not a parameter integrated in the ABP and, thus, represents a potential confounding factor in its interpretation (Bejder et al., 2017; Lobigs et al., 2018c; Krumm and Faiss, 2021).

Thereafter, the parameter of Hb_mass_ was commonly used in the literature to illustrate hematological variations to reduce PV shifts and to explain possible increases in performance (Zelenkova et al., 2019). The correlation between changes in HGB [%] and the hypoxic dose might be related to similar results reported by Garvican-Lewis et al. (2016), who identified a linear correlation between Hb_mass_ [%] and the hypoxic dose of 55% (*R*^2^ = 0.546). Thus, 55% of the variation in Hb_mass_ [%] and km.h might be explained by the hypoxic dose. The residual variance (remaining 45%) should be explained by other parameters, such as the training loads, other environmental conditions, the athletes' initial blood parameters (HGB values and iron status), and the individual response to hypoxic exposure. Although the fact that our relation between HGB levels and the hypoxic dose did not consider the PV, unlike the relation reported by Garvican-Lewis et al. (2016), both relations are similar and confirm the concomitant evolution of those parameters.

Many authors observed an increase in Hb_mass_, following 7–10 days of altitude exposure (Garvican et al., 2012; Beidleman et al., 2017; Clark et al., 2017). A previous study has also shown that the beginning of the increase in Hb_mass_ varied between individuals and required at least 4 days in most subjects and up to 12 days in one subject (Siebenmann et al., 2015). This delayed response has been explained by Lewis (1989) as the maturation time of an erythrocyte of 5–7 days. However, increased s-EPO levels are not necessarily associated with an increase in Hb_mass_. In fact, Gore et al. (2006) did not find any evidence of accelerated erythropoiesis by measuring indirect markers, such as HGB, RET% or MCV, and even reported an increase in s-EPO levels after 4 weeks of IHE. The 21 h/day spent under normoxic conditions for 5 days plus the whole weekend might cancel the short-term effect of IHE (Gore et al., 2006). Furthermore, some studies suggested a possible link between the initial Hb_mass_ value and the individual variability in the increase in Hb_mass_ caused by LHTL camps (Robach and Lundby, 2012). In this latter study, a negative correlation was observed between baseline values and the maximal increase in Hb_mass_ (r = 0.86, *p* < 0.01). However, Hauser et al. (2016) did not provide any proof of a correlation (r = 0.02, *p* = 0.92) between the baseline Hb_mass_ value (in g) and the relative increase in absolute % Hb_mass_, suggesting that well-trained endurance athletes, even with a high initial Hb_mass_ value, might also increase their Hb_mass_ above the 95% CL (e.g., 1,024–1,075 g, +5%). Siebenmann et al. (2015) also did not identify any correlation between the initial value of Hb_mass_ and final increases (r = −0.09, *p* = 0.82). The same authors observed a marked increase in the probability for Hb_mass_ to reach a plateau after 20–24 days. Finally, the highest rate of increase in Hb_mass_, which occurred after 14.9 ± 5.2 days, was 4.04 ± 1.02 g/day (Siebenmann et al., 2015). A large interindividual variation remained for the increase in Hb_mass_, where Rasmussen et al. (2013) identified that a 5% increase occurred from Day 17 to Day 41 at 3,500 m.

#### Effect on Off-Hr

Because the Off-hr is defined as inversely proportional to RET% variations, it decreased in the first 200 km.hr, with the increase in the number of immature red blood cells. Then, it returned to the baseline value as soon as RET% started to decrease and HGB levels began to increase (Lobigs et al., 2018b). The Off-hr would continue to increase above baseline levels if the hypoxic dose exceeded 1,000 km.hr (Lobigs et al., 2018b). At this point, the increase in Off-hr was due to its proportional relationship with HGB modifications. Ashenden et al. (2003) identified 2 of 11 elite cyclists, who lived and trained at 2,690 m and whose Off-hr exceeded the reference range set at 99.9% of specificity (at this time, the so-called 1 in the 1,000 Off model). A similar situation was observed by Bonne et al. (2014), who registered three Olympic-level swimmers, with Off-hr values exceeding the reference limits with an average increase of 17 ± 12 units at the end of the hypoxic exposure. Although most of the values remained within the reference ranges, an increase in Off-hr always occurred after altitude exposure compared to pre-altitude exposure in the selected studies [8.5, 11.6, and 21.3% in the studies by Garvican-Lewis et al. (2018), Wachsmuth et al. (2012), and Bonne et al. (2014), respectively].

#### Post-Altitude Training Effect on Biomarkers

When returning to the sea level, adaptations to the normoxic environment include decreases in HGB and RET%, while Off-hr increases progressively return to baseline values (Lobigs et al., 2018b). Indeed, the HGB increased during altitude camps, but once the athlete left the hypoxic environment and returned to the sea level, the HGB levels started to decrease progressively until reaching baseline values within 2–3 weeks (Ashenden et al., 2003; Bonne et al., 2014; Lobigs et al., 2018b). The decrease in HGB levels was partially attributed to fast PV expansion when returning to the sea level (Boning et al., 1997). As stated above, PV contraction and hemoconcentration occur from the 1st days of altitude exposure; however, the renin-angiotensin-aldosterone axis is reactivated when returning to the sea level. These changes result in hemodilution and return the PV to baseline values, inducing this decrease in HGB levels (Siebenmann et al., 2015). Moreover, RET% decreased below the baseline after 5 to 9 days of hypoxic exposure (Bonne et al., 2014). This might be induced by neocytolysis activation noted by several authors to selectively eliminate the youngest erythrocytes that had formed during stress-induced erythropoiesis under hypoxic conditions and were no longer needed when the athlete returned to normoxic conditions (Rice and Alfrey, 2005; Mairbaurl, 2013; Ploszczyca et al., 2018). This mechanism remains unclear but might be initiated by the sudden decrease in s-EPO when returning to the sea level as well as an altered antioxidant capacity of new erythrocytes (Mairbaurl, 2018). Again, interindividual variability in hypoxic exposure influenced the decrease in HGB levels after altitude exposure, such that some athletes reached baseline values within 3 days (Heinicke et al., 2005). Additionally, the colder environmental climate experienced under HH conditions might help explain the different decreases in HGB levels compared to NH conditions (Wachsmuth et al., 2012). After the LHTH camp, the decrease in HGB levels was explained differently by hemodilution despite a total increase in Hb_mass_ during the camp (Ploszczyca et al., 2018). The increased Hb_mass_ persisted after returning to the sea level and was still +3.4% at 20 days after altitude exposure compared to baseline values in the study by Gore et al. (2013). Considering the rapid return of Hb_mass_ and HGB levels to baseline values, the interval between the return to the sea level after altitude training and a competition should be extremely short (1–3 days) (Heinicke et al., 2005) or after 2–3 weeks (Schmidt and Prommer, 2005; Siebenmann et al., 2015). Finally, Turner et al. (2017) suggested implementing 2 h/day of NH exposure at 4,200 m or higher to attenuate the decrease in s-EPO levels and maintain a high Hb_mass_ after returning to the sea level. Therefore, careful planning before, during, and after an altitude camp, particularly before a competition, is needed when the objective is to improve performance related to the hematological adaptations of the camp.

Regarding the Off-hr, results obtained after altitude exposure were more complex to analyze than the HGB and RET% values. Off-hr was expected to increase after hypoxic exposure, as described by Lobigs et al. (2018b). Indeed, 3 weeks after altitude exposure, Off-hr should peak again with decelerated erythropoiesis (on-phase) when HGB levels decreased and RET% started to increase again (Gore et al., 2003). Ashenden et al. (2003) illustrate that, after 14 days at 2,690 m, an ~17-unit group mean increase in Off-hr occurs. For the LHTH group in this study (lived and trained at 1,690 m), the postaltitude Off-hr increased by ~12 units after 9 days. In the study by Wachsmuth et al. (2012), the Off-hr of elite swimmers living and training at 2,320 m increased by 9.5 and 8.7 units for women and men, respectively, 3-4 weeks after returning to the sea level. Finally, Bonne et al. (2014) identified an Off-hr increase of ~14 units in some athletes at 7 days after 3-4 weeks of LHTL at 2,130–3,094 m (+21.25% between pre-1 and post-7). However, further research focusing on the postaltitude hematological response is needed to better understand the mechanisms and their dynamics.

#### Decrease in s-EPO Levels After Altitude Training

The decrease in hemoglobin levels might be explained by two main factors. First, when the subjects returned to the sea level for postaltitude measures, s-EPO levels started to decrease significantly below the baseline. Two selected studies illustrated −26 and −34% (HH and NH conditions, respectively) decreases in s-EPO levels on Day post-1 (Saugy et al., 2016b), and −26% 2 days after the subjects returned to the sea level (Clark et al., 2009). These decreases in s-EPO levels were not as substantial as those observed by Rice et al. (2001), who reported a decrease of 80% after the hypoxic exposure. In LHTH camps, the decrease in s-EPO levels from pre- to post-exposure measurements is well described in the literature. Conversely, this decrease was not as high as that observed after LHTL camps, because of the “oscillating” protocols proposed with several descents per day to lower altitudes (Garvican et al., 2012). This phenomenon was even less important in LHTL under NH conditions than under HH conditions. Indeed, daily shifts between hypoxia and normoxia occurred more often under NH conditions than under HH conditions [7 vs. 2 in the study of Saugy et al. (2014a)]. The second reason is the onset of neocytolysis described above. Different time courses of the decrease in Hb_mass_ may be explained by the magnitude of the decrease in s-EPO levels (Garvican et al., 2012). Nonetheless, the interindividual responses of the decrease in Hb_mass_ when returning to the sea level after hypoxic exposure, according to the magnitude of the decrease in s-EPO levels, remained high (Garvican et al., 2007).

#### Iron Supplementation and the ABP

Erythroferrone (ERFE) has been highlighted as a crucial endocrine regulator of erythropoiesis and iron metabolism (Emrich et al., 2021). Erythropoiesis induced by altitude exposure through s-EPO secretion stimulates the production of ERFE, leading to a decrease in the plasma hepcidin concentration, which will, in turn, disturb iron homeostasis (Robach et al., 2009; Badenhorst et al., 2014). This disturbance will result in iron absorption and iron store release (Goetze et al., 2013), a phenomenon that is well perceived at altitude with the decrease in serum ferritin levels (Roberts and Smith, 1992; Stray-Gundersen et al., 2001; Heinicke et al., 2005; Goetze et al., 2013). Hence, high-iron levels must be maintained to assure an improved blood oxygen-carrying capacity through new RBC production. In their study, Garvican-Lewis et al. (2018) identified 11 of 34 athletes whose biomarkers values surpassed the 99% specificity threshold limit of the ABP after adapted iron supplementation (3 weeks at 3,000 m, ~882 km.h). Thus, the effects of iron and ferritin statuses before, during, and after altitude training on the ABP should be considered by experts who evaluate abnormal blood profiles.

### Erythropoietin and Blood Parameters

#### General Considerations and Overview

Quickly after the marketing of rhEPO in 1989, many studies started to investigate its effect on performance (Berglund and Ekblom, 1991; Trinh et al., 2020). Indeed, if hypoxic exposure is an allowed method to increase an RBC count, the use of erythropoiesis-stimulating agents, such as rhEPO, became the most common method to increase the RBC mass in a nonnatural and prohibited manner. Special attention should be given to whether the subjects of these studies were mostly recreationally active participants, or simply healthy volunteers. However, no clinical study using rhEPO agents involving elite athletes has been conducted, and one should be aware of this distinction between the subject's levels and the elite population when interpreting the results.

**Figure 3:**
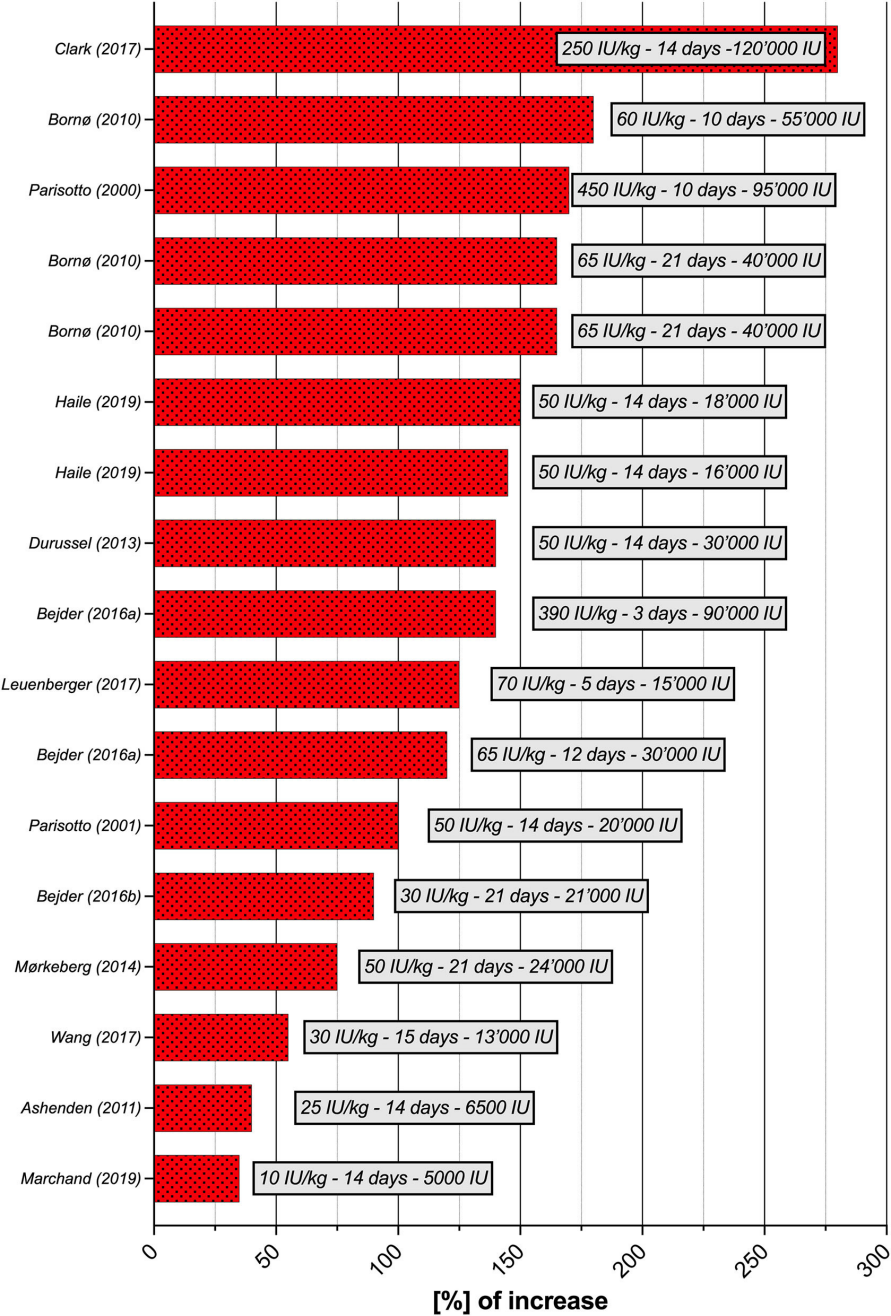
Increase in [%] of RET% per study (*n* = 13), with doses per body weight (IU/kg), duration and total dose at the time of the greatest increase in RET%. RET%, reticulocyte percentage.

Seventeen studies were selected for the EPO part of the literature search. Although the experimental parameters of each study are very different, the aim is to present a situational analysis of research on rhEPO injections that have been conducted, with the aim of helping to the interpret ABP results for the anti-doping community. Therefore, Table 2 provides an overview of the most significant results according to the study designs used. The doses per body weight ranged from 10 IU/kg to 450 IU/kg (average of 103.4 ± 130.2 IU/kg) and total doses at the end of the study varied from 5,000 to 120,000 IU (average of 51,458.8 ± 31,283.4 IU). The maximal percent increase in RET% occurred in the study by Clark et al. (2017), with 280% on Day 14 (body weight dose of 250 IU/kg), and the minimal percent increase was recorded in the study by Marchand et al. (2019), with 4% on Day 14 (body weight dose of 10 IU/kg). The maximal increase in RET% was observed after an average duration of 13.9 ± 5.2 days of treatment. The mean increase in RET% was 128 ± 60.3%. Figure 3 illustrates the significant increase in RET% reported in 13 articles retrieved from the literature search. The erythropoietin dose per body weight, duration of the maximal increase in RET%, and total dose at the end of the study are also presented. In 12 studies, the increase in HGB levels was significant and ranged from 4 (Marchand et al., 2019) to 19% (Ashenden et al., 2006; Clark et al., 2017), with a mean of 11.9 ± 4.9%. A determination coefficient of R^2^ = 0.77 between the percent increase in HGB levels (%) and the total dose of EPO (IU) injected was calculated. The sensitivity of the ABP was mentioned in 6 studies and ranged from 0 to 94%. Finally, only five studies did not supply their subjects with any of the following substances: elemental iron, intravenous iron, ferrous fumarate, vitamin C, vitamin B12 or ferrous sulfate.

**Table 2:** Summary of the main physiological parameters collected in the 17 EPO studies of the literature search.

**N**°****	**Autor + year**	**Subjects [n]**	**Supplement**	**EPO dose [IU/kg]**	**Total dose [IU]**	**Dose injected at highest effect on RET% [IU]**	**Frequency of injections**	**HGB increase [%]**	**RET increase [%]**	**Abnormal ABP markers [%]**
1	Ashenden et al. (2006)	2	100 mg elemental iron daily	260	105'000	-	Six high doses over 2 weeks and 9 micro doses over 3 weeks	18	-	-
2	Ashenden et al. (2011)	10	105 mg elemental iron daily	25	40'000	6,500	Two per week for 8 weeks	6.4	40	0
3	Bejder et al. (2016a)	16	no	65	35'000	30'000	Every other day for 2 weeks (7 doses)	NS	120	31 during, and 13, 11 days after
		16		390	90'000	90'000	Three consecutive days	NS	140	21 immediately after and 33, 8 days after
4	Bejder et al. (2016b)	20	100 mg elemental iron daily	30	24'000	21'000	Three high doses per week for 3 weeks + 2 micro doses	7.6	90	-
5	Birkeland et al. (2000)	10	270 mg iron daily	200	60'000	-	Three per week during 30 days	14	-	-
6	Borno et al. (2010)	8	-	65	50'000	40'000	Four per week during 2 weeks + 1 per week during 2 weeks	-	165	58
		8		63	50'000	40'000	Four per week during 2 weeks + 1 per week during 2 weeks	-	165	-
		8	100 mg elemental iron daily	60	90'000	55'000	Four per week during 2 weeks, 3 consecutive days on week 3, 1 per week for 7 weeks	-	180	-
7	Clark et al. (2017)	8	100 mg IV iron	250	120'000	120'000	Six doses over 2 weeks	17–19	280	75 immediately after and 19, 3 weeks after
		8	100 mg IV iron	250	130'000	120'000	Six doses over 2 weeks + 9 micro doses over 3 weeks	-	-	-
8	Durussel et al. (2013)	19	100 mg elemental iron daily	50	60'000	30'000	Four per week for 4 weeks	16	140	-
9	Haile et al. (2019)	20	100 mg elemental iron daily	50	45'000	16'000	Every other day for 4 weeks	13	145	17/18
		19	100 mg elemental iron daily	50	60'000	18'000	Every other day for 4 weeks	17	150	-
10	Heuberger et al. (2019)	23	200 mg ferrous fumarate + 50 mg ascorbic acid	80	48'000	-	Once a week for 8 weeks	12	-	-
11	Leuenberger et al. (2017)	6	no	70–75	15'000	15'000	Single doses on days 1–3–5	NS	125	-
12	Marchand et al. (2019)	8	105 mg elemental iron, 500 mg vit. C and B12 daily	10	5,000	5,000	Three per week during 2 weeks	4	35	-
13	Martin et al. (2016)	20	100 mg elemental iron daily	30	22'000	-	Three per week during 3 weeks + 2 micro doses after 10- and 14-days washout	9	−30	-
		12	100 mg elemental iron daily	11	1,800	-	Two micro doses	-	-	-
14	Morkeberg et al. (2014)	7	100 mg elemental iron daily	50	25'000	23'400	Two per week during 3 weeks + 2 micro doses on week 4	7.6	75	-
15	Parisotto et al. (2000)	24	105 mg elemental iron daily	300–600	95'000	95'000	Two, three or four injections over 10 days	-	170	-
16	Parisotto et al. (2001)	41	IV iron	50	40'000	20'000	Three per week over 25 days	-	100	-
17	Wang et al. (2017)	14	350 mg ferrous sulfate	20–40	30'800	12'600	Twice a week over 7 weeks	-	55	-

*Only the highest HGB and RET% increases are represented. EPO, erythropoietin; RET%, reticulocyte percentage; HGB, concentration of hemoglobin; ABP, athlete biological passport*.

**Figure 4:**
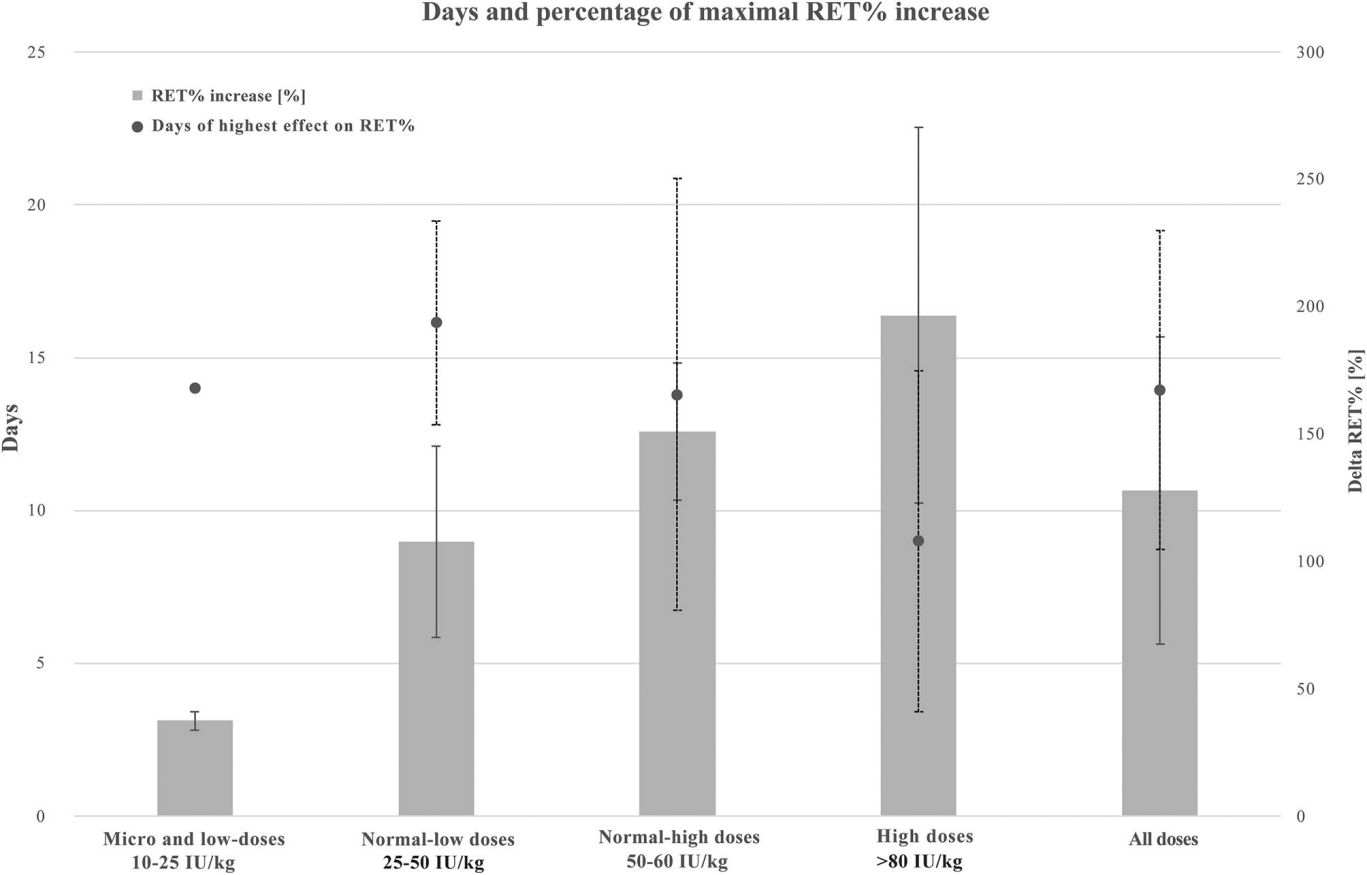
Combined graphic illustrating maximal RET% increase and average days of highest RET% increase when using micro and low (10–25 IU/kg, *n* = 2), normal-low (25–50 IU 7 kg, *n* = 7), normal-high (50–80 IU/kg, *n* = 5), high (250–450 IU/kg, *n* = 3), and all the doses of rhEPO. Values are presented in average and standard deviation (SD). A solid line for RET% increase ± SD and a dotted line for days of highest effect on RET% ± SD. RET%, reticulocyte percentage.

Figure 4 presents the maximal increase in RET% according to four categories of rhEPO doses and all doses combined. Average and standard deviation (SD) increases in RET% when protocols used micro and low, normal-low, normal-high, high, and all doses were 37.5 ± 3.5%, 107.9 ± 37.5%, 151. ± 26.8%, 196.7 ± 73.7%, and 127.9 ± 60.3%, respectively. Regarding the number of days where the highest increase in RET% was observed, the average and SD results were 14. ± 0.0, 16.1 ± 3.3, 13.8 ± 7., 9. ± 5.6, and 13.9 ± 5.2 days, respectively.

#### Evolution of Hematological Parameters During the “Injection Phase”

Recombinant human EPO, similar to hypoxic exposure, stimulates erythropoiesis by increasing s-EPO levels, RET%, HGB levels, and Hb_mass_ (Parisotto et al., 2000).

The reticulocyte percentage has been identified as the most sensitive parameter for the detection of rhEPO misuse during a period with frequent injections (Borno et al., 2010). A previous study has identified RET% evolution (on-phase) after the administration of a single high dose of rhEPO (epoetin alpha EPREX, 150–300 UI/kg) and showed that RET% started to increase after 36 h, reached its maximal increase after 3–4 days, and normalized within 7 days (Major et al., 1994). rhEPO may increase the lifespan of RET from the baseline value of 1.7 to 3.4 days (Krzyzanski and Perez-Ruixo, 2007). Thus, treatment with rhEPO appears to exert a dual effect on RET%: increasing the production of reticulocytes in the bone marrow and prolonging the time during which red cells remain immature. Clark et al. (2017) also reported that 2 weeks of injections of high rhEPO doses increased RET% values by 3- to 4-fold compared to baseline values. The reticulocyte percentage is expected to peak during the first 2 weeks of rhEPO treatment (Morkeberg et al., 2014; Siebenmann et al., 2015). In this literature search, an average of 14 ± 5 days was required to reach a maximal RET% increase of 128 ± 60%. These results confirmed the time estimate previously established, but the increase in the level compared to baseline values was lower. However, we considered studies using both micro and high doses. The high-dose regimen required less time, only 9 ± 5.6 days, to reach the maximal RET% increase (196.7 ± 73.7%) than the other regimens. Nevertheless, only three studies were included in this category (Parisotto et al., 2000; Ashenden et al., 2006; Clark et al., 2017). Thus, these results should be interpreted with caution. Previous studies have also documented increases in HGB levels, HCT, Hb_mass_, and erythrocyte volume. These increases were combined with a decrease in PV, such that the total blood volume remained stable. Hence, blood viscosity increased (Morkeberg et al., 2014; Siebenmann et al., 2015). This blood thickening phenomenon might lead to dangerous health issues for athletes and explains why many study protocols carefully monitored the increase in HGB levels and HCT during the injection phase, and established a predetermined safety HCT limit [55% in the study by Haile et al. (2019), for example]. During the 2nd week, when the production of new erythrocytes was highly stimulated, Bejder et al. (2016b) revealed an expected Off-hr peak (off-phase). In fact, this period coincided with the peak of the increase in RET% and preceded the peak increase in HGB levels.

After RET% started to increase, HGB levels also began to increase, following rhEPO injections. Previous studies have shown that HGB levels should peak in the 1st week after rhEPO treatment (Audran et al., 1999; Bejder et al., 2016b). This review illustrated increases ranging from 4 to 19% (average, 11.9 ± 4.9%) between the pre- and post-tests. These results were consistent with a previous study, which showed an increase in HGB levels of 11% after 20 IU/kg rhEPO injections three times per week for 6 weeks (Berglund and Ekblom, 1991). Another highlight of the present review is the positive correlation between an increase in the HGB percentage and the total dose of rhEPO injected. In fact, 77% of the increase in the HGB levels was explained by the total dose of rhEPO injected during the study. Interesting comparisons were performed between two studies with the same total dose obtained at the end of their respective protocols. For example, Birkeland et al. (2000), Durussel et al. (2013), and Haile et al. (2019) proposed similar protocols with rhEPO doses of 50–67 IU/kg administered three to four times a week for 4 weeks. They all reached a total dose of 60,000 IU of EPO. The final increase in HGB-level increases was similar at 16, 17, and 14%, respectively. However, when comparing the study by Haile et al. (2019), with two studies that proposed the highest doses of rhEPO per body mass [260 and 250 IU/kg) and total mass (105,000 and 120,000 IU for Ashenden et al. (2006), Clark et al. (2017), respectively], the increase in HGB levels was only 1% higher (i.e., 17 vs. 18%). Thus, even with a smaller dose of rhEPO per body mass, a similar increase in HGB levels might be reached with less possibility of detection by the anti-doping tests. However, further research should be conducted to clarify this hypothesis.

Finally, Hb_mass_ was increased in several selected studies, for example, by 19.7% after the administration of four doses per week of 50 IU/kg rhEPO for 4 weeks (Durussel et al., 2013), 18.4% after three injections per week of 250 IU/kg epoetin beta for 2 weeks (Clark et al., 2017) or 10.5% after two injections per week of micro-doses of rhEPO (10–40 IU/kg) for 12 weeks (Ashenden et al., 2011). Similar to altitude exposure, the increase in Hb_mass_ is considered the primary mechanism responsible for the ergogenic effects of rhEPO use. Interestingly, a 1-g increase in Hb_mass_ produces a change in VO2*max* of 4 ml/min−1 (Ashenden et al., 2001; Schmidt and Prommer, 2005).

#### Post-Injection Phase

Similar to changes that occur following altitude exposure, RET% entered the off phase and decreased below baseline levels after rhEPO injections (Pottgiesser et al., 2012), with the lowest values recorded in the 2nd week of washout (Clark et al., 2017). Because of the decrease in RET%, the Off-hr increased compared with baseline values. Then, 3 to 4 weeks after the last rhEPO injection, Off-hr noticeably decreased again with decelerated erythropoiesis (on-phase), while HGB levels decreased and RET% started to increase again (Gore et al., 2003). The main differences between the effects of hypoxic exposure and rhEPO injections on hematological parameters in the post-experimental phase were the duration for which those effects were maintained and the amplitude of the changes. Hemoglobin levels and Hb_mass_, which increased to 17–19% and 18.4%, respectively, compared to the baseline, have been shown to remain elevated 4 weeks after the last high-dose injections by Clark et al. (2017). Thus, the effects of rhEPO injections appear to last longer than those of altitude exposures.

#### Iron Supplementation

A previous study has documented the importance of iron supplementation while injecting ESAs (Major et al., 1997). Iron administration, particularly when injected IV, increased the efficiency of ESA. Similar to altitude exposure, the use of rhEPO will increase the need for iron to correctly stimulate the production of new erythrocytes, and iron supplementation results in increased ferritin levels in athletes. However, even with this iron complementation, the availability of this element is quickly reduced (Clark et al., 2017), and some subjects may not benefit from the effects of the rhEPO injection. This situation might be explained by ESA hypo responsiveness (or resistance). The clinical definition of this pathology is the limited increase in HGB levels even after the administration of unusually high doses of ESA caused by noncompliance, absolute or functional iron deficiency and inflammation (Johnson et al., 2007). A participant in the study by Clark et al. (2017) presented a similar case of hypo-responsiveness where iron supplementation (a single IV dose) 2 weeks before the injection of a high dose of rhEPO did not increase his HGB level and RET% as expected. In this case, the authors suggested that the iron supply was too low to support the RBC production needs. Indeed, this subject presented a low initial serum ferritin level (16 umol/l) and transferrin saturation (32%). Nonetheless, in the 2nd week after rhEPO treatment, the adaptive model of the ABP still flagged suspicious and abnormal RET% and Off-hr for this subject (Clark et al., 2017).

#### rhEPO and the ABP

Compared to hypoxic exposure, the literature search showed that the selected biomarkers of the hematological module of the ABP were more efficient to flag rhEPO administration. RET% is more sensitive than HGB and Off-hr after a boosting phase with high doses of rhEPO (94% and 88% suspicious and abnormal RET% values, respectively). Even during the washout period, RET% sensitivity remained high and flagged atypical ABP profiles. Conversely, Off-hr sensitivity was lower during the washout period of the high-dose protocol (37.5% suspicious and 13% abnormal) and during the 1st week of the micro-dose phase. Ultimately, 100% of the ABP profiles were considered as having suspicious Off-hr (among which 75% were identified as abnormal) 2 weeks after the administration of the high dose in the HIGH and COMB groups in the study by Clark et al. (2017). Bejder et al. (2020) also showed that RET% might enhance the sensitivity of the ABP when combined with HGB levels and Off-hr. This author focused on examining the hypothesis that RET% should be considered as a primary biomarker to indirectly detect rhEPO misuses. Some studies showed that not all of the subjects who were administered rhEPO were detected by the ABP software or by purpose-built spreadsheets similar to the adaptive model [see, for example, Heuberger et al. (2019)]. The results from the study by Haile et al. (2019) showed a potential false negative ABP (never exceeding one of the biomarker limits during and after rhEPO administration) for 1 of 18 profiles. This subject may be less responsive to rhEPO administration than the others and, therefore, may not improve his performance even with the usage of rhEPO.

#### Micro-Doses of rhEPO and the ABP

As mentioned above, athletes switched to rhEPO micro-doses due to the appearance of EPO tests and indirect methods to detect the use of rhEPO. According to a previous study, micro-dose of rhEPO increased Hb_mass_ up to 10% (Ashenden et al., 2011), where a 5% increase in Hb_mass_ is already sufficient to improve sea-level performance (Robach and Lundby, 2012). Increasing Hb_mass_ is not the only advantage of treatment with rhEPO micro-doses. In fact, they have also been used to induce only small variations in blood parameters to mask obvious hematological changes observed after blood manipulations, such as autologous blood transfusions or the use of larger rhEPO doses. In fact, blood transfusions are known to reduce the production of RET due to the artificial increase in the red cell mass (Schumacher et al., 2012). The same situation occurred during the off phase of RET% in the washout period after rhEPO injection due to the feedback control of EPO production (Jelkmann, 2016). Injecting micro-doses of rhEPO might then induce a small RET% increase, which is sufficient to mask suspicious ABP profiles but not sufficient to be detected by direct urine or blood tests (Ashenden et al., 2011). In their study, Ashenden et al. (2011) showed that using low doses prevented the detection of all 10 athletes who received 20 to 30 IU/kg rhEPO two times a week for 8 weeks. However, the lack of collection of post-rhEPO samples in this study, as well as the specificity level of the ABP software set to 99.9%, which is beyond the normal value of 99%, might have influenced the poor performance of the adaptative model. In another way, micro-dosing might also maintain a high HGB level for a longer period. Once the HGB level is increased by the administration of high doses of rhEPO, only micro-doses or less-frequent injections of the drug are needed to maintain a certain concentration (Lundby et al., 2007). The reduced probability of being caught by direct detection was supported by Ashenden et al. (2006), where intravenous injection of 10 IU/kg rhEPO in the evening was considered difficult to detect the next day with conventional detection methods.

Recently, Martin et al. (2021) have shown that the detection window of rhEPO in urine was prolonged up to 48 h and 72 h compared to serum/plasma. In this study, blood detection using either the improved IEF-PAGE or SDS-PAGE methods, and new criteria for IEF-PAGE allowed us to identify 100% of rhEPO micro-doses (Binocrit epoetin alpha, 10 IU/kg, three times a week for 2 weeks) 24 h after the last injection and 64 to 82% after 48 h. For urine detection, the SDS-PAGE method flagged as suspicious samples with 100% of rhEPO micro-doses 48 h after the last injection and 91% after 72 h (Martin et al., 2021).

This short detection window of rhEPO was exploited in a previous study with a special doping protocol (Clark et al., 2017). Athletes first received high-dose injections of rhEPO in a “boosting phase” followed by a “maintenance phase” in which micro-doses of rhEPO were injected to maintain the beneficial effects of the substance without being detected by the anti-doping tests. Different blood parameters, such as Hb_mass_, HCT, HGB levels, RBC counts, and erythrocyte volume (EV), were monitored. The aim of this method was to maintain high HGB levels while masking the effects expected after the administration of a last high dose of rhEPO. All the blood parameters, except EV, increased and remained high after 3–4 weeks. Interestingly, ABP sensitivity remained high, and outliers were flagged by the adaptative model of blood-doping detection (Clark et al., 2017). During the micro-dose phase and the 1st week after micro-doses were administered, Off-hr and RET% were sensitive to the previous boosting phase (Clark et al., 2017). These results illustrated the possibility for athletes to intentionally use micro-doses of rhEPO to leverage the short detection window (which depends on the route of administration, the dosage injected, the type of rhEPO injected, etc.) of the substance and profit from the physiological and performance enhancement with a reduced probability of being caught (Clark et al., 2017).

Determining the perfect timing to identify a doped athlete with known direct methods is extremely difficult and will rarely lead to anti-doping rule violations (ADRV) in athletes using micro-dosing regimens (Martin et al., 2016). However, suspicious ABP profiles remained extremely useful for the anti-doping process as they allow further targeted direct and/or indirect tests (blood and/or urine) to be conducted. These results could be flagged as an atypical passport, finding (ATPF) by the ABP adaptative model in ADAMS, and then considered as a “likely doping” case by the initial expert, and, finally, judged as an ADRV by the panel of experts.

## Conclusion

The role of the ABP is to help anti-doping experts identify athletes who transgressed the rules and to protect those who play true. The complexity regarding hematological and functional adaptations to altitude exposure and rhEPO injections increases the difficulty of identifying doped athletes by experts. Therefore, biomarkers that differentiate changes induced by hypoxia and rhEPO administration have been identified, and physiological adaptations of these biomarkers, during, and after hypoxia and rhEPO intake must be screened to improve the current ABP (Sutehall et al., 2019). By performing a literature search, the present study aimed to assemble and identify physiological effects on blood biomarkers of the ABP induced by two external factors: altitude and EPO. The objective was to provide keys to understand and interpret the ABP hematological module affected by hypoxic exposure or rhEPO misuse. In fact, the literature search allowed us to understand the temporal effects of different hypoxic exposures and rhEPO injections on several hematological parameters.

On one hand, the hypoxic conditions, time of exposure, training loads, and number of measures must be examined when interpreting those physiological variations, and the hypoxic dose should not be considered alone. This literature review also suggested that PV shifts should not be underestimated. Indeed, their effects on the HGB level through hemoconcentration in the first 2 days of hypoxic exposure (increase in HGB levels and Off-hr, but not RET% and Hb_mass_), or hemodilution during several days of maximal effort (decrease in HGB levels and Off-hr, but increases in RET% and Hb_mass_) have been well established by the scientific community. Iron levels must also be evaluated to optimize a positive erythropoiesis response, following hypoxic exposure.

The order of physiological events under hypoxic conditions could be enumerated as follows: an increase in s-EPO levels 1–2 days after the beginning of hypoxic exposure, an increase in RET% in the first 300 km.h that is associated with an Off-hr decrease in the first 200 km.h, the start of increases in HGB levels and Hb_mass_ between 4 and 12 days (HGB levels and Hb_mass_ correlated with the hypoxic dose at 60 and 55%, respectively), with a plateau of the increased HGB levels of 0.94 g/dl observed at 1,000 km.hr. The s-EPO levels started to decrease beginning on the 1st day after returning to the sea level, followed by a decrease in RET% during the 1st week. Neocytolysis then induced a decrease in the RBC count and, subsequently, Hb_mass_, which remained high for up to 3 weeks post-exposure. The decrease in hemoglobin levels started 3 days after hypoxia exposure or took more time, up to 3 weeks. Thus, the Off-hr returned to the baseline between 9 days and 4 weeks. This long period is problematic, as WADA guidelines currently require specific information about only 2 weeks before a blood or urine test.

Most importantly, the significance of interindividual variability has been highlighted, showing that, even if average modifications are reported, not every subject would positively react to hypoxic exposure, and the range of variation among those who respond might prevent the identification of strong cause-effect relationships. Taken together, these elements support the hypothesis that hypoxic exposure is an important confounding factor in the ABP and its interpretation.

On the other hand, we aimed to better understand the effects of rhEPO use on hematological parameters, and RET% was considered the most sensitive parameter and should be carefully measured in any studies that are designed to test rhEPO doping protocols to help the anti-doping research field. A mean increase in RET% of 128 ± 60% was identified through the selected studies using rhEPO. The smallest increase in RET% occurred when low micro-doses of rhEPO were administered, and high doses of rhEPO led to the greatest increase in RET%. The number of days for a maximal increase in RET% was between 9 and 19. This period coincided with the first negative Off-hr peak, where RET% increased substantially due to stimulated erythropoiesis, but HGB levels had not yet increased. The hemoglobin concentration showed an average increase of 11.9 ± 4.9% and a strong positive correlation with the total dose of rhEPO. Finally, two studies identified in the literature search registered high increases in Hb_mass_ of 18.4 and 19.7% (Durussel et al., 2013; Clark et al., 2017). When rhEPO administration was terminated, studies measured a decreased in RET% during the first 2 weeks, while HGB levels and Hb_mass_ remained high for 3 to 4 weeks. Thus, the Off-hr increased and sometimes exceeded the baseline value.

In addition, micro-doses of rhEPO induced significant increases in various hematological parameters, leading to performance enhancement and reducing the detection window with a direct test (urine and/or blood). Finally, some protocols have shown that the combination of a boosting phase (with high doses of rhEPO), followed by a maintenance phase (with micro-doses), might mask the obvious effects that follow rhEPO use while maintaining the hematological benefits and the performance improvement.

However, the sensitivity of the ABP remained high, and atypical passports were flagged by the adaptative model. False negative passports occurred in some studies, which might be explained by ESA hypo responsiveness. Similar to hypoxia exposure, the strategy of supplementing subjects with iron during rhEPO injection is particularly important and must not be underestimated when interpreting hematological results.

Primary biomarkers of the ABP, namely, HGB levels and Off-hr, were considered sufficiently efficient to indirectly detect rhEPO doping when high doses were administered. The reticulocyte percentage also showed important variations and might be useful as a stand-alone primary biomarker of the ABP.

The external factors of altitude and EPO illustrated similar temporal changes, RET% increase during the first 2 weeks, followed by a subsequent increase in HGB levels and a decrease in the Off-hr before returning to baseline levels. After the experimental phase, RET% decreased in the first 2 weeks, and the Off-hr started to increase compared to baseline levels. The altitude studies showed increases of 7 ± 3.9%, 69.8 ± 139.9%, and 4.7 ± 1.6% in HGB levels, RET%, and Hb_mass_, respectively (for IHE, LHTH, and LHTL only). For the rhEPO studies, the mean increases in HGB, RET%, and Hb_mass_ were 11.9 ± 4.9%, 119.2 ± 69.4%, and 19 ± 1%, respectively. Increases in the levels of those three parameters were, respectively, 1.7, 1.7, and four times higher in rhEPO studies than in altitude studies. These variations illustrated that hypoxia exposure, as well as rhEPO use, disrupted the natural variability of the physiological parameters of the ABP and might, therefore, alter the sensitivity and/or specificity of the adaptative model.

Notably, significant increases in hematological parameters were not observed in all studies (see Tables 1, 2). Moreover, the wide interindividual variability of changes in hematological parameters following hypoxic exposure or rhEPO administration should be considered. The main topic highlighted by this study is that the average dose used in the experimental protocols did not reflect the reality of the doping process that athletes might use. In fact, micro-doses are more likely to be used by doped athletes since the detection window is short when direct blood or urine tests are performed (Martin et al., 2021). In addition, micro-doses potentially induce a physiological evolution similar to that induced by hypoxia exposure (Sutehall et al., 2019). Unfortunately, indirect detection of rhEPO micro-doses by the ABP remains limited (Ashenden et al., 2011). Consequently, the efficiency of the ABP mainly depends on the timing and targeting strategy for sample collection.

In addition, the ability of anti-doping authorities to understand the effects of various confounding and external factors on the physiological parameters of the athlete is crucial to better monitor and test those parameters. Further methods of detection, such as emerging “omics” approaches, including the analysis of changes in gene expression by rhEPO administration (Wang et al., 2017), or the monitoring of hepcidin and erythroferrone levels to detect autologous blood transfusion or rhEPO use (Leuenberger et al., 2017; Andersen et al., 2022), must be supported. The effects of hematological variations on performance, which are briefly discussed in this study, should also be considered. A similar tool to the ABP, with longitudinal analyses of performance data, might be useful as a complement to the existing modules (Hopker et al., 2018).

## Author Contributions

JS and TS conceived the project and contributed to the collection of data. JS, TS, and FB interpreted the data. JS wrote the first draft of the manuscript. All authors contributed to revising the manuscript and expressed their approval of the final submitted version.

## Funding

Open access funding provided by University of Lausanne.

## Conflict of Interest

The authors declare that the research was conducted in the absence of any commercial or financial relationships that could be construed as a potential conflict of interest.

## Publisher's Note

All claims expressed in this article are solely those of the authors and do not necessarily represent those of their affiliated organizations, or those of the publisher, the editors and the reviewers. Any product that may be evaluated in this article, or claim that may be made by its manufacturer, is not guaranteed or endorsed by the publisher.
